# 76. Hospitalized Patients Have Microbiome Dysbiosis Linked to Post-Discharge Infection

**DOI:** 10.1093/ofid/ofaf695.027

**Published:** 2026-01-11

**Authors:** Chad Hinkle, Huaiying Lin, Shanna Banogan, Jackelyn Cantoral, Caroline Jadczak, Ashley Sidebottom, Victoria Burgo, Sabrina Imam, Matthew Odenwald, Emily Landon, Eric Pamer, Christopher Lehmann

**Affiliations:** University of Chicago Medicine, Chicago, IL; University of Chicago, Bellevue, Washington; University of Chicago, Bellevue, Washington; University of Chicago, Bellevue, Washington; University of Chicago, Bellevue, Washington; University of Chicago, Bellevue, Washington; University of Chicago, Bellevue, Washington; University of Chicago, Bellevue, Washington; University of Chicago, Bellevue, Washington; University of Chicago, Bellevue, Washington; University of Chicago, Bellevue, Washington

## Abstract

**Background:**

A healthy gut microbiome offers protection against pathogenic bacteria, such as multidrug-resistant organisms (MDROs), *Enterococcus* and *Enterobacterales*. Expansion of these organisms is a harbinger for invasive infection and could contribute to the spread of MDROs. Acute care hospitals account for the majority of MDRO infections nationally. To test whether hospitalized patients develop pathogenic expansion within the microbiome, we examined the gut microbiome and metabolome of hospitalized patients and compared them to post-discharge infection.

**Figure:**
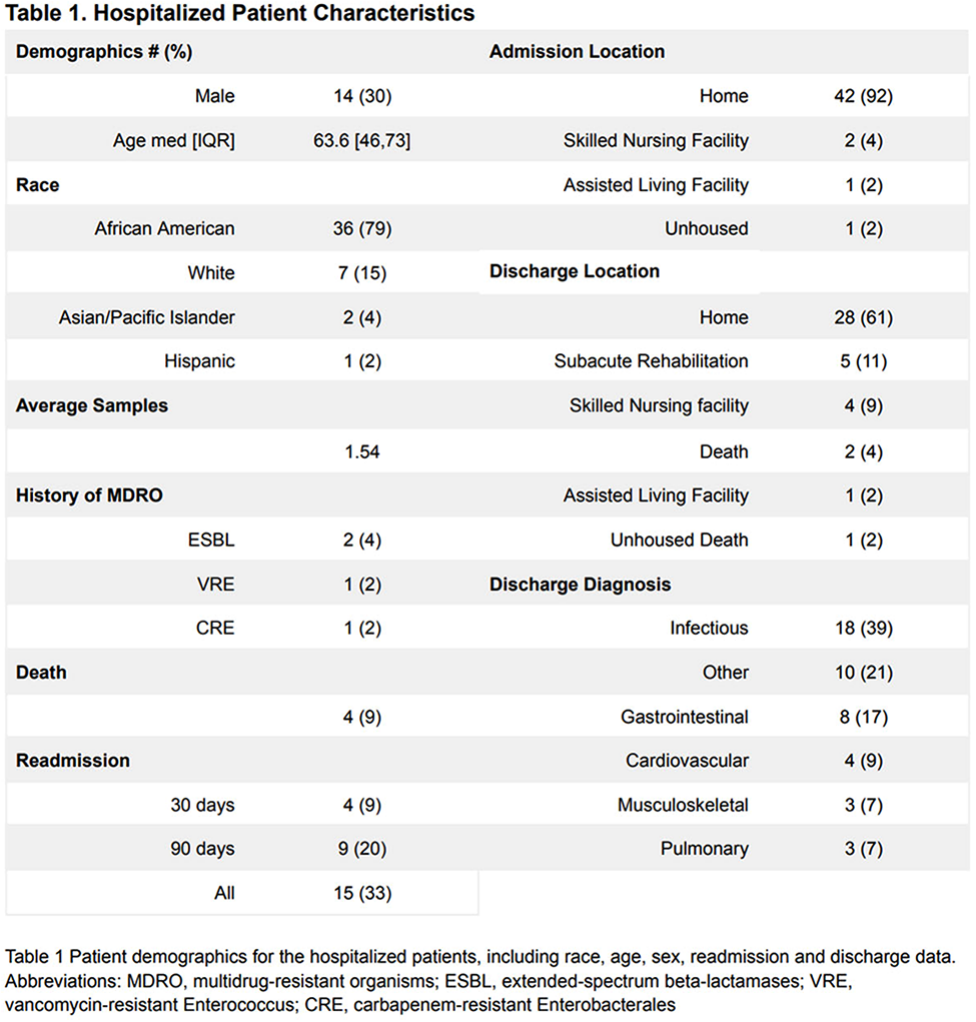

**Figure:**
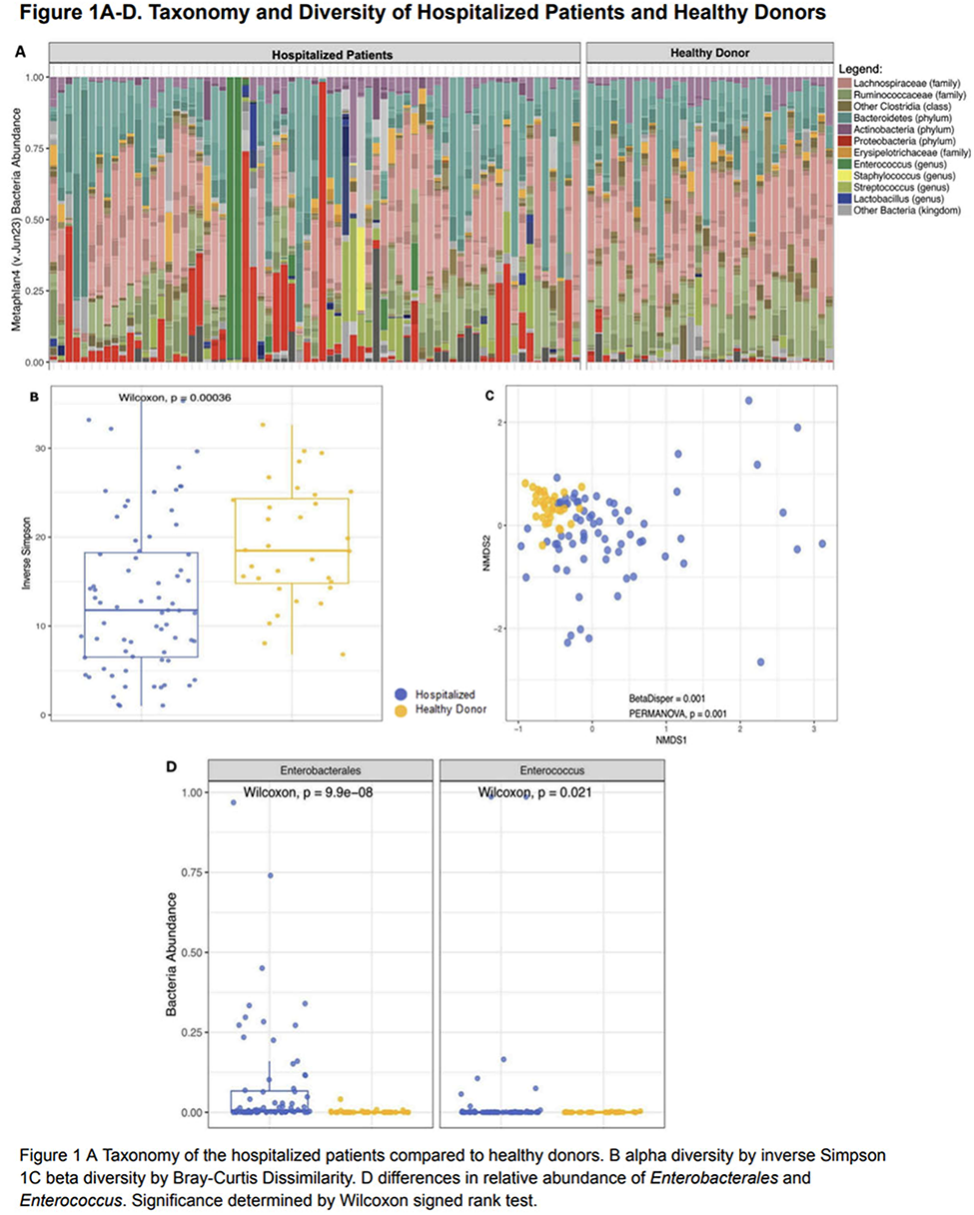

**Methods:**

In a prospective, observational study we collected stool samples from hospitalized patients on a single unit that approximates a general inpatient population. We performed shotgun metagenomic sequencing and quantitative GC and LC-MS metabolite measurement on each sample. We examined the differences in microbiota diversity and composition to healthy donors and compared discharge microbiome composition to post-discharge infection.

**Figure:**
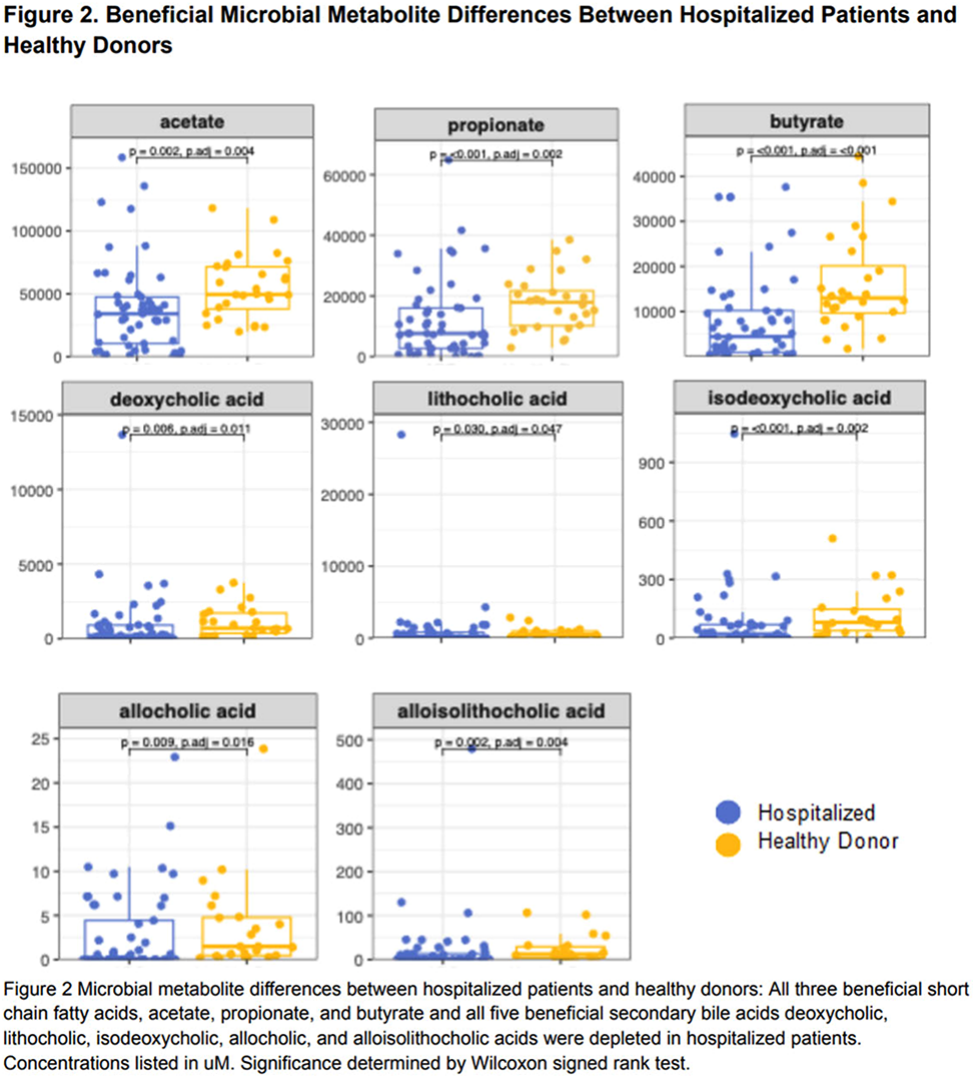

**Figure:**
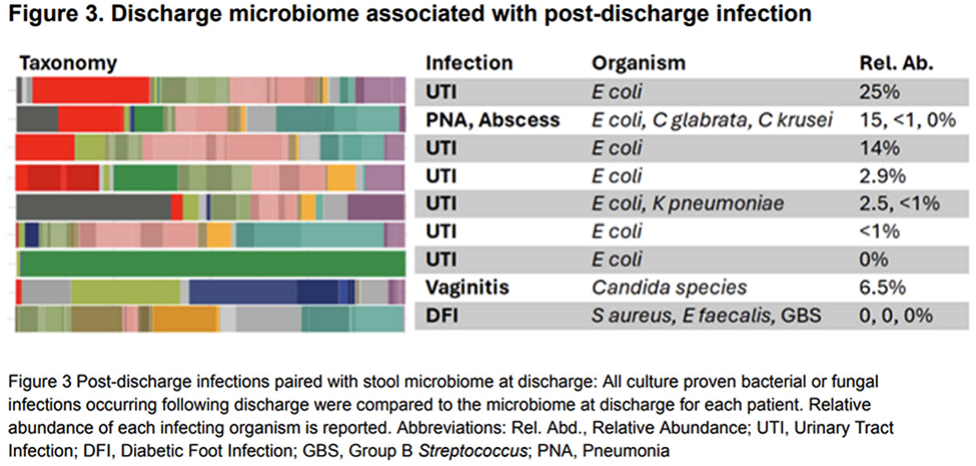

**Results:**

A total of 71 samples were collected from 46 hospitalized patients (Table 1). Compared to 32 healthy donors, hospitalized patients had lower diversity, compositional changes and expansion of both *Enterobacterales* and *Enterococcus* in 17 (23%) and 6 (8%) samples, respectively (Figure 1). Beneficial microbe-derived short chain fatty acids and secondary bile acids were also reduced (Figure 2). Among hospitalized patients that developed post-discharge infection, the discharge microbiome had 9 (64%) of the infecting organisms (Figure 3). For infections caused by *Enterobacterales*, 7 (88%) were detected in the discharge microbiome. *E.coli* expanded >2.5% in 5 (71%) patients with *E.coli* infection. *Candida* was found in 2 (66%) discharge stool samples that developed a *Candida* infection.

**Conclusion:**

Hospitalized patients have a reduction in microbiome diversity, beneficial microbial metabolites, and pathologic expansion of *Enterobacterales* and *Enterococcus* species. Expansion at the time of discharge was associated with post-discharge infection. Gut microbiome expansion in this population could be responsible for the rise of MDROs. Therapies to restore microbiome composition and function could be used to combat MDRO infections.

**Disclosures:**

All Authors: No reported disclosures

